# The Marine Mammal Class II Major Histocompatibility Complex Organization

**DOI:** 10.3389/fimmu.2019.00696

**Published:** 2019-04-04

**Authors:** André Luiz Alves de Sá, Breanna Breaux, Tibério Cesar Tortola Burlamaqui, Thaddeus Charles Deiss, Leonardo Sena, Michael Frederick Criscitiello, Maria Paula Cruz Schneider

**Affiliations:** ^1^Laboratory of Applied Genetics, Socio-Environmental and Water Resources Institute, Federal Rural University of the Amazon, Belém, Brazil; ^2^Laboratory of Genomics and Biotechnology, Biological Sciences Institute, Federal University of Pará, Belém, Brazil; ^3^Comparative Immunogenetics Laboratory, Department of Veterinary Pathobiology, College of Veterinary Medicine and Biomedical Sciences, Texas A&M University, College Station, TX, United States; ^4^Center for Technological Innovation, Evandro Chagas Institute, Belém, Brazil; ^5^Center of Biodiversity Advanced Studies, Biological Sciences Institute, Federal University of Pará, Belém, Brazil

**Keywords:** molecular evolution, genomics, marine mammals, manatee, MHC, immunogenetics, pinnipeds, cetaceans

## Abstract

Sirenians share with cetaceans and pinnipeds several convergent traits selected for the aquatic lifestyle. Living in water poses new challenges not only for locomotion and feeding but also for combating new pathogens, which may render the immune system one of the best tools aquatic mammals have for dealing with aquatic microbial threats. So far, only cetaceans have had their class II Major Histocompatibility Complex (MHC) organization characterized, despite the importance of MHC genes for adaptive immune responses. This study aims to characterize the organization of the marine mammal class II MHC using publicly available genomes. We located class II sequences in the genomes of one sirenian, four pinnipeds and eight cetaceans using NCBI-BLAST and reannotated the sequences using local BLAST search with exon and intron libraries. Scaffolds containing class II sequences were compared using dotplot analysis and introns were used for phylogenetic analysis. The manatee class II region shares overall synteny with other mammals, however most *DR* loci were translocated from the canonical location, past the extended class II region. Detailed analysis of the genomes of closely related taxa revealed that this presumed translocation is shared with all other living afrotherians. Other presumptive chromosome rearrangements in Afrotheria are the deletion of *DQ* loci in Afrosoricida and deletion of *DP* in *E. telfairi*. Pinnipeds share the main features of dog MHC: lack of a functional pair of *DPA/DPB* genes and inverted *DRB* locus between *DQ* and *DO* subregions. All cetaceans share the Cetartiodactyla inversion separating class II genes into two subregions: class IIa, with *DR* and *DQ* genes, and class IIb, with non-classic genes and a *DRB* pseudogene. These results point to three distinct and unheralded class II MHC structures in marine mammals: one canonical organization but lacking *DP* genes in pinnipeds; one bearing an inversion separating IIa and IIb subregions lacking *DP* genes found in cetaceans; and one with a translocation separating the most diverse class II gene from the MHC found in afrotherians and presumptive functional *DR, DQ*, and *DP* genes. Future functional research will reveal how these aquatic mammals cope with pathogen pressures with these divergent MHC organizations.

## Introduction

The transition from terrestrial to aquatic habitat has occurred in several terrestrial vertebrate lineages. In mammals, early after the Cretaceous period, independent ancestral lineages of afrotherian, cetartiodactyl, and carnivore would begin their path to return to aquatic environments which would lead to current sirenian, cetacean, and pinniped species of marine mammals. Those three lineages have further undergone adaptive radiation and their descendants are found in both oceanic and freshwater habitats.

The order *Sirenia* is represented by one species of dugong (*Dugong dugon*) and three species of manatees (*Trichechus manatus, T. senegalensis*, and *T. inunguis*), all of them exclusively herbivorous and whose closest living relatives are the elephants. The order Cetacea have approximately 89 species divided into two suborders: Odontoceti (toothed whales) and Mysticeti (baleen whales), and is closely related to hippopotamuses. Pinnipedia comprises a carnivore suborder with three families (Otaridae, Phocidae, and Odobenidae) of around 34 species of aquatic fin-footed mammals (seal, sea-lions, and walrus), closely related to bears and musteloids (e.g., raccoons and skunks), which are still dependent on the land to live, in contrast to sirenians and cetaceans, which are totally adapted to the aquatic environment. Aquatic mammals share several convergent traits selected for fresh water and marine habitats, including morphologic and genetic traits (1–4).

Living in water poses new challenges not only for locomotion and feeding but also for combating new pathogens. How the three major independent aquatic mammal lineages just detailed dealt with the genetic constraints of their ancestry and to what extent their recent history in similar habitats led to convergent evolution in their immune system is not clear. Several marine mammals lack major predators in the adult phase, so infectious disease may be an important cause of mortality (5). This may make the immune system of these aquatic lineages particularly important for their fitness and fecundity. Compared to their terrestrial relatives, marine mammals face distinct diversity of pathogens, disease ecology, and epidemiology (6–8), which may create distinct selective pressures on immune genetic systems, including the Major Histocompatibility Complex (MHC).

The MHC encodes many immune (and many non-immune) genes, canonically divided in class I, II, and III regions in vertebrates. The class II region includes classical (e.g., *DR, DQ, DP*) MHC, non-classical (e.g., *DO, DM*) MHC, antigen processing (e.g., *TAP, PSMB*) and other genes. Classical class II alpha and beta genes encode a protein heterodimer that presents antigens for T lymphocytes to detect infections and other danger signals. Classical MHC genes are highly polymorphic, confer resistance or susceptibility to diseases, and may be used as genetic markers for species conservation (9, 10). Several studies have reported the diversity of class II genes in cetaceans (11–19) and pinnipeds (5, 20–25). Past evidence also showed that class II MHC genes may be important genetic markers for survival in a seal species (5). Despite its proposed importance for marine mammals, so far only a representative of cetacean has had their class II MHC organization characterized (26). Therefore, we aimed to compare the genomic organization and evolution of the MHC class II region in sirenians, cetaceans, and pinnipeds, using genome assemblies from representatives of these groups available in public databases. We also included other mammals from different eutherian lineages for a better understanding of the evolution of marine mammals and the eutherian class II MHC region.

## Materials and Methods

### MHC Class II Genes Identification and Reannotation

The marine mammals investigated in this research were: the sirenian Florida manatee (*Trichechus manatus latirostris)*; the cetaceans minke whale (*Balaenoptera acutorostrata scammoni*), sperm whale (*Physeter catodon*), baiji (*Lipotes vexillifer*), beluga whale (*Delphinapterus leucas*), finless porpoise (*Neophocaena asiaeorientalis*), bottlenose dolphin (*Tursiops truncatus*), Pacific white-sided dolphin (*Lagenorhynchus obliquens*), and killer whale (*Orcinus orca*); and the pinnipeds walrus (*Odobenus rosmarus*), Northern fur seal (*Callorhinus ursinus*), Hawaiian monk seal (*Neomonachus schauinslandi*), and Weddell seal (*Leptonychotes wedelli*). We included in the analysis other mammals as outgroups and representatives of other major eutherian branches. A summary of the assembly reports from each analyzed species is provided in Supplementary File S1.

Preliminary search on the NCBI database identified annotated MHC class II genes in the genomes of cetaceans, afrotherians, and pinnipeds. All predicted mRNA gene sequences were aligned to their human homologs. We selected presumptive well-annotated classical genes based on the presence of full-length sequences, presence of all exons, and no evidence of pseudogene misidentification (presence of stop codons and lack of homology in any exons). Those predicted genes and their human homologs were used to perform megablast and discontiguous megablast searches in the genomes of marine mammals and other mammals representative of the main eutherian branches. Gene references used were: *DMA*, NM_006120.3; *DMB*, NM_002118.4; *DOA*, NM_002119.3; *DOB*, NM_002120.3; *DRA*, NM_019111.4 and XM_007951302.1; *DRB*, NM_002124.3 and XM_003423461.2; *DPA*, NM_001242525.1 and XM_006882197.1; *DPB*, NM_002121.5 and XM_012559980.1; *DQA*, NM_002122.3 and XM_003421050.1; *DQB*, NM_001243961.1.

We checked all predicted class II gene and pseudogene sequences for proper annotation using Geneious 9 (27). MAFFT (28) alignments with the predicted coding sequence were used to check for missing or poorly annotated exons. We constructed local BLAST libraries containing exons and introns for each gene and performed blast on scaffolds containing class II sequences. Nomenclature used for class II genes of non-model species included a prefix formed by the first two letters of the genus and species (i.e., *Loxodonta africana*, Loaf; *Trichechus manatus*, Trma; *Orycteropus afer*, Oraf; *Elephantulus edwardii*, Eled; *Chrysochloris asiatica*, Chas; *Echinops telfairi*, Ecte; *Dasypus novemcintus*, Dano; *B. acutorostrata*, Baac; *D. leucas*; Dele; *L. vexillifer*, Live; *N. asiaeorientalis*, Neas; *O. orca*, Oror; *L. obliquidens*, Laob; *T. truncates*, Tutr; *P. catodon*, Phca; *C. ursinus*, Caur; *N. schauinslandi*, Nesc; *L. weddelli*, Lewe; *O. rosmarus*, Odro; *Pteropus alecto*, Ptal; *Equus caballus*, Eqca) (29, 30); BoLA, DLA, H2 and HLA were used for bovine, dog, mouse and human genes, respectively.

Predicted coding sequences with no stop codons or frameshift mutations were presumed to be functional and annotated as genes (including incomplete coding sequence due to assembly gaps); sequences with at least one stop codon or frameshift mutations were annotated as pseudogenes. Thus, for clarity, in this manuscript “locus/loci” will be used when broadly referring to sequences from a gene family, including both presumed genes and pseudogenes; “gene” will be used when referring only to presumed functional sequences; and “pseudogene” will be used for presumed non-functional sequences. New annotations and reannotation CDS, as well as the summary of the loci used in this study are provided here as Supplementary Files S2,S3.

### Comparative Genomics Analysis

To construct dot plot graphs, the manatee, Northern fur seal and sperm whale were chosen as representatives of the main marine mammal branches. Scaffolds containing class II genes were first submitted to RepeatMasker (31) and resulting masking files were used along with sequence and gene annotations on PipMaker (32). For the manatee, scaffolds covering the class II MHC region were concatenated. Part of the annotations and sequences from the extended class II region were removed to provide a better view of the identity of key regions.

### Phylogenetic Analysis

We constructed phylogenetic trees using the exons and introns from classical MHC class II genes and pseudogenes (when they could be accurately determined). We chose to use only loci located in scaffolds that allowed us to determine their location. Introns were separately aligned using MAFFT online service (33) and the alignments cleaned in GBlocks (34) under default settings and allowing gaps in all sequences. Intron alignments were then concatenated for the rest of the analysis. Exons were also aligned using MAFFT. Best-fit partition scheme and corresponding nucleotide substitution model was checked on PartitionFinder (35); each intron was discriminated for the search of all possible partition schemes and each codon position was treated as a partition. Maximum likelihood trees were constructed in CIPRES (36) using RAxML (37), with 1,000 bootstrap iterations. All phylogenetic tests were performed in triplicate. Trees were constructed on iTOL (38). Exon phylogenies did not change the main conclusions of this study, therefore we present only intron phylogenies since they are more comprehensive.

## Results

### The Marine Mammal Genomes and Class II MHC Synteny

All marine mammal genomes in our analysis were *de novo* assembled using varying assembly methods (Supplementary File S1). The manatee genome was made using Illumina Hi-seq technology with a 150x coverage; pinniped genomes were also made using Illumina reads with coverage ranging from 27.44x to 200x; and cetacean genomes were made using Illumina or BGISEQ-500 technology, with coverage ranging from 35.68x to 248x (Supplementary File S1).

The manatee class II sequences were distributed over eight scaffolds, while other marine mammals had their class II MHC distributed on 1–3 scaffolds (Table 1). Overall, we were able to locate one copy of each non-classical gene in all marine mammals, whereas the classical genes varied across taxa. MHC class II genes showed conservation in sequence length and number of exons when compared to human homologs, although many entries were only partial due to gaps in the genome or difficulty in determining exon boundaries.

**Table 1:** Number^a^ of MHC class II genes and pseudogenes in each marine mammal scaffolds.

**Species**	**Scaffold**	**DRA**	**DRB**	**DQA**	**DQB**	**DPA**	**DPB**	**DOA**	**DOB**	**DMA**	**DMB**	**DYA/DYB**
*T. manatus*	NW_004444001	0	0 (1ψ)	0	0	0	0	0	0	0	0	0
	NW_004444197	0	0	0	0	0 (1ψ)	0 (4ψ)	0	0	0	0	0
	NW_004444318	0	0	0	0 (2ψ)	1	1	1	1	1	1	0
	NW_004444391	1	0	1	1 (1ψ)	0	0	0	0	0	0	0
	NW_004444463	0	0 (1ψ)	0	0	0	0	0	0	0	0	0
	NW_004444511	0	0	0	0	0	1	0	0	0	0	0
	NW_004444627	0	0	0	0	1	0	0	0	0	0	0
	NW_004446990	0	0 (1ψ)	0	0	0	0	0	0	0	0	0
*B. acutorostrata*	NW_006728570	0	0 (1ψ)	0	0	0	0 (1ψ)	1	1	1	1	0
	NW_006732678	1	1 (1ψ)	0	0	0	0	0	0	0	0	0
	NW_006731889	0	1	0	0	0	0	0	0	0	0	0
*D. leucas*	NW_019160881	1	2 (1ψ)	1	1	0	0	1	1	1	1	0
*L. vexillifer*	NW_006778796	0	0 (1ψ)	1	1	0	0	0	0	0	0	0
	NW_006787800	0	0 (1ψ)	0	0	0	0	1	1	1	1	0
	NW_006786873	1	1 (1ψ)	0	0	0	0	0	0	0	0	0
*N. asiaeorientalis*	NW_020174277	1	1 (1ψ)	1	1	0	0	0	0	0	0	0
	NW_020175393	0	0 (1ψ)	0	0	0	0	1	1	1	1	0
*O. orca*	NW_004438437	0	0 (1ψ)	0	0	0	0	1	1	1	1	0
	NW_004438672	1	2	1	1	0	0	0	0	0	0	0
*L. obliquidens*	NW_020837975	1	1 (2ψ)	1	1	0	0	1	1	1	1	0
*T. truncatus*	NW_017842945	0	0 (1ψ)	0	0	0	0	0	1	1	1	0
	NW_017844288	1	2 (1ψ)	1	1	0	0	0	0	0	0	0
*P. catodon*	NW_019873557	1	2 (1ψ)	1	1	0	0	1	1	1	1	0
*C. ursinus*	NW_020313370	1	1 (1ψ)	1	1	0 (1ψ)	0 (2ψ)	1	1	1	1	0
	NW_020319034	1	0	0	0	0	0	0	0	0	0	0
	NW_020321179	1	0	0	0	0	0	0	0	0	0	0
*N. schauinslandi*	NW_018734297	0	0	1	1	0 (1ψ)	0 (1ψ)	1	1	1	1	0
	NW_018734368	0 (1ψ)	1	0	0	0	0	0	0	0	0	0
*L. weddellii*	NW_006383774	1	1	0	0	0	0	0	0	0	0	0
	NW_006383968	0	1	1	1	0 (1ψ)	1	0	1	1	1	0
	NW_006386795	0	0	0	0	0	0	1	0	0	0	0
*O. rosmarus*	NW_004450609	0	1	0	0	0 (1ψ)	0 (1ψ)	1	1	1	1	0
	NW_004450757	2	1 (1ψ)	0	0	0	0	0	0	0	0	0
	NW_004452682	0	1	1	1	0	0	0	0	0	0	0

a *The number of presumptive functional genes are outside of brackets, and number of pseudogenes (ψ) between brackets*.

**Figure 1:**
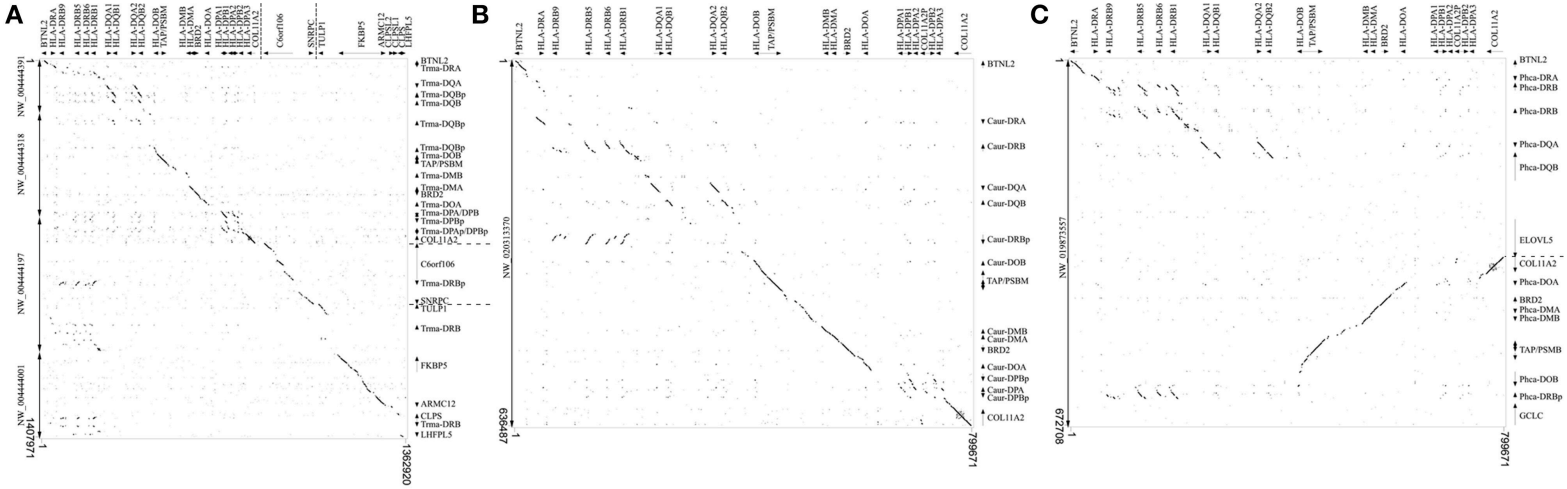
Dot plot analyses of **(A)** *Trichechus manatus*, **(B)** *Callorhinus ursinus*, and **(C)** *Physeter catodon* scaffolds containing MHC class II genes, compared to the same region of the human genome. Human gene annotations are shown on the top of the graphs. For clarity purposes, extended class II region was reduced to include only key regions containing class II sequences; dashed lines on the annotation represent gaps. *T. manatus* sequences were in different scaffolds and were therefore concatenated for this analysis.

We chose a representative of each marine mammal lineage to construct dot plot graphs against the human MHC. The manatee class II region maintain the overall synteny compared to human, but all *DRB* loci are located after the extended class II region (Figure 1A). The Northern fur seal class II region also have the main features of the human MHC class II organization; however it lacks conservation in the *DP* subregion and possesses an inverted *DRB* pseudogene between its *DQ* and *DO* subregions (Figure 1B). The sperm whale class II region is divided in two subregions due to an inversion separating the *DR* and *DQ* genes from the non-classic genes (Figure 1C); the cetacean also lacks identity in the *DP* subregion and possesses a *DRB* pseudogene between *DOB* and *GCLC* (Figure 1C).

### Main Features of the Marine Mammal MHC Class II Region

**Figure 2:**
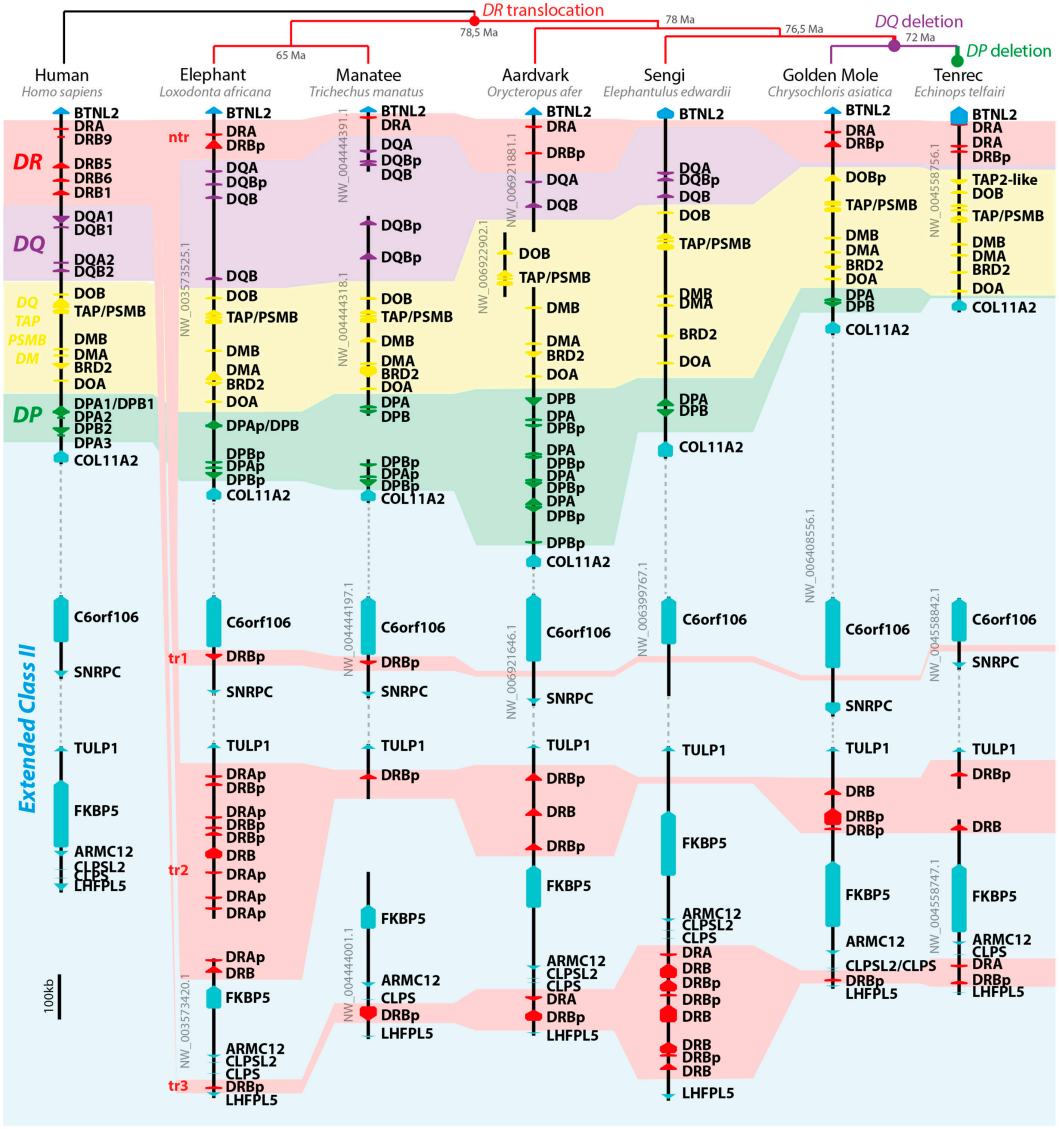
Model of afrotherian class II MHC evolution. On the top of image, the afrotherian phylogeny and divergence time (Ma, million years before present) proposed by Springer et al. (39) and presumptive evolutionary events leading to current afrotherian MHC structure. On the left the human class II MHC region is depicted as an outgroup and model of the mammalian genome organization. Dashed lines represent regions of the scaffolds excluded for clarity purposes, which are not to scale. Arrows represent genes and pseudogenes (shown as “p” in the end of the gene's name). Only informative scaffolds of the MHC structure are displayed. In color, a schematic view of class II loci helps to understand the evolution of class II loci (*DR*—red; *DQ*—purple; *DP*—green; *DO/TAP/PSMB/DM* —yellow). *TAP/PSMB* represents *TAP1, TAP2, PSMB8, PSMB9*.

Due to the fragmentation of the manatee class II region in distinct scaffolds, we turned our attention to other afrotherians to understand their organization. The genomes investigated here were all sequenced and assembled by the Broad Institute. *L*. a*fricana* was the first sequenced *afrotherian* genome, assembled with Sanger reads; other *afrotherian* genomes were sequenced by NGS Illumina Hi-seq technology. All genomes were *de novo* assembled, with coverage ranging from 44x to 150x for the Illumina assembled genomes (Supplementary File S1). *C. asiatica* and *E. edwardii* have all class II loci in the same scaffold, evidence that class II sequences lie in the same chromosome. All analyzed afrotherian share the presumptive translocation of *DR* loci (Figure 2); this translocation was not found in other boreoeutherian or xenarthran genomes analyzed here (data not shown). Other presumptive chromosomal rearrangements were the deletion of *DQ* in the ancestor of *Afrosoricida* (i.e., *C. asiatica* and *E. telfairi*) and deletion of *DP* in *E. telfairi* (Figure 2).

**Figure 3:**
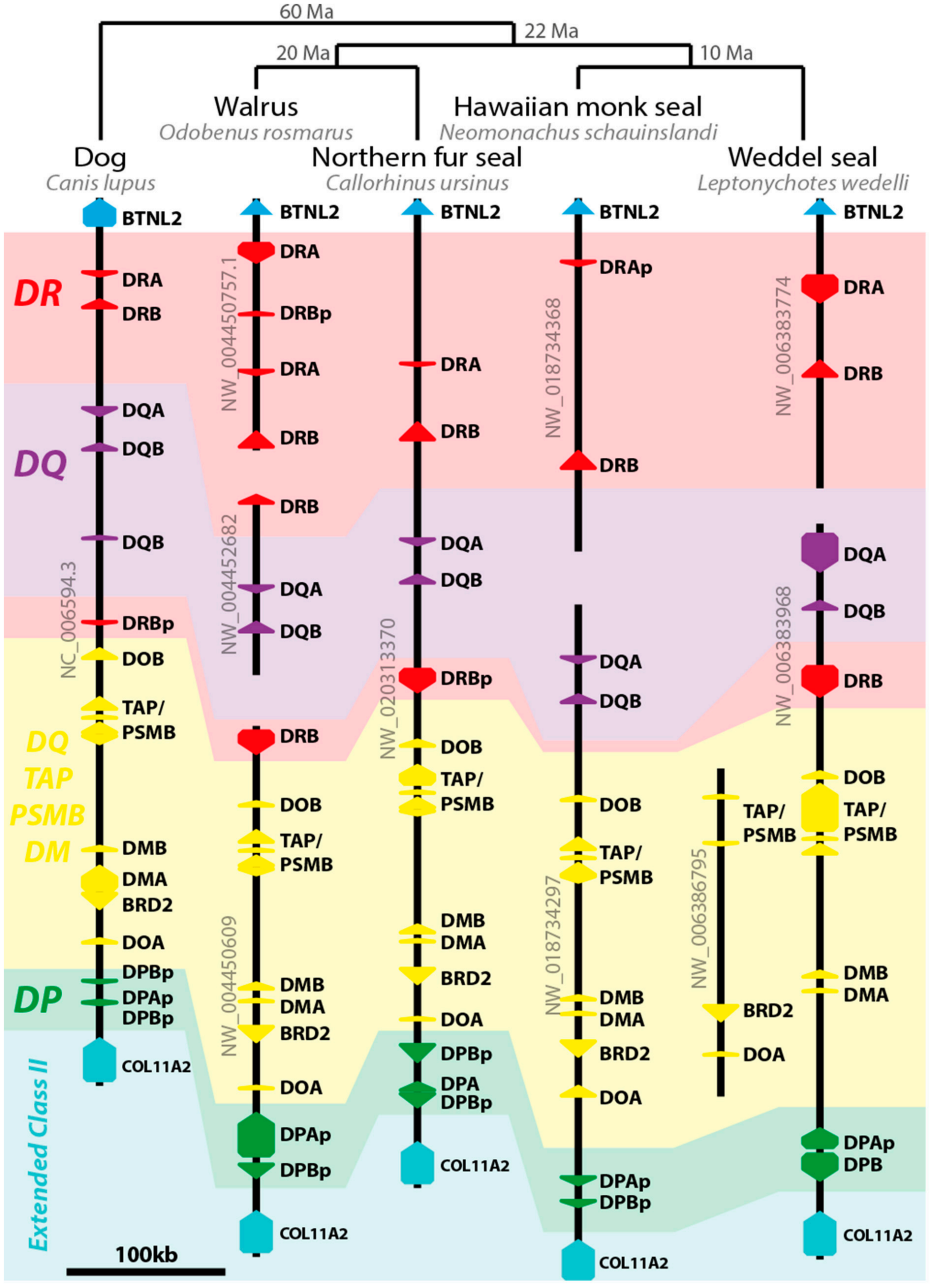
Model of pinniped class II MHC evolution. On the top of image, the pinniped phylogeny and divergence time (Ma, million years before present) proposed by Nyakatura and Bininda-Emonds (40). On the left, dog class II MHC region is depicted as an outgroup and model of the Carnivora genome organization. Arrows represent genes and pseudogenes (shown as “p” in the end of the gene's name). Only informative scaffolds of the MHC structure are displayed. In color, a schematic view of class II loci helps to understand the evolution of class II loci (*DR*—red; *DQ*—purple; *DP*—green; *DO/TAP/PSMB/DM*—yellow). *TAP/PSMB* represents *TAP1, TAP2, PSMB8, PSMB9*.

The pinniped class II region has the same composition found in the dog DLA (Figure 3). Like the Northern fur seal, walrus and Weddell seal also possess *DRB* loci between *DOB* and *DQB*. Pinniped genomes have varied numbers of *DR* loci but a single pair of presumed *DQ* genes. Most *DP* loci seem to be pseudogenes in pinnipeds (Figure 3).

**Figure 4:**
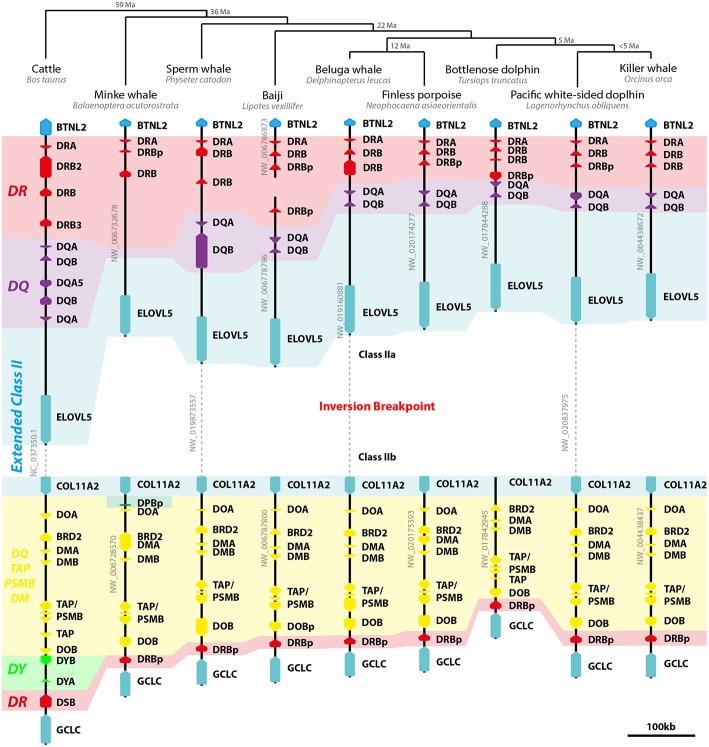
Model of cetacean class II MHC evolution. On the top of image, the Cetartiodactyla phylogeny and divergence time (Ma, million years before present) proposed by Zurano et al. (41). On the left, cattle class II MHC region is depicted as an outgroup and model of the terrestrial Cetartiodactyla genome organization. Dashed lines represent regions of the scaffolds excluded for clarity purposes, which are not to scale. Arrows represent genes and pseudogenes (shown as “p” in the end of the gene's name). Only informative scaffolds of the MHC structure are displayed. In color, a schematic view of class II loci helps to understand the evolution of class II loci (*DR*—red; *DQ*—purple; *DP*—green; *DY*—light green; *DO/TAP/PSMB/DM*—yellow). *TAP/PSMB* represents *TAP1, TAP2, PSMB8, PSMB9*.

All cetaceans share with terrestrial Cetartiodactyla the inversion separating class II genes in two subregions: IIa, including *DR* and *DQ* loci; and IIb, including non-classic genes (Figure 4). Cetaceans have one *DRA* gene and up to three *DRB* loci in class IIa and a presumed *DRB* pseudogene next to *DOB* on class IIb region. Despite lying in the same location occupied by *DYB* and *DSB* in cattle, those sequences share a higher homology with *DRB* exons and introns and therefore were annotated as such. Most cetaceans have only one pair of *DQ* genes, whereas no *DQ* loci was found in the minke whale genome. Like cattle, cetaceans seem to have lost *DP* loci altogether, with only remnants of a *DPB* pseudogene found in the minke whale class IIb region. No *DY* loci was found in any cetacean (Figure 4).

### Non-classical Class II and Antigen Processing Genes

Overall, non-classical genes were already annotated in the genomes analyzed here, but some entries needed a refined prediction of exon boundaries. The gene content and organization across marine mammals is highly conserved, as found in other mammals. Notably, *D. leucas* and *E. telfairi DOB* had to be separated from the *TAP2* gene annotations. *L. vexillifer* and *C. asiatica DOB* have a stop codon at exon 5, therefore were annotated as presumed pseudogene despite being the only predicted *DOB* locus in their genomes. Protein alignments showed conservation of exon sizes, with most differences related to missing exons due to gaps in assembly (Supplementary Files S4–S7).

### Classical Class II Genes

#### *DR* Loci

Most *DR* loci had to be reannotated, especially the small exons 5 and 6 from *DRB*. Protein alignments of *DR* genes are provided in Supplementary Files S8, S9. In the three orders of marine mammals we were able to find *DRA* and *DRB* genes, despite manatee having most of its sequences outside the canonical class II region. The translocation of *DR* loci in *Afrotheria* split sequences into four subregions (Figure 2): within the canonical class II region (not translocated, “nt”), between *C6orf106* and *SNRPC* (translocation 1, “tr1”; ~2.3 Mb distant from nt), between *TULP1* and *FKBP5* (tr2; mean ~3.2 Mb distant from nt), and between *CLPS* and *LHFPL5* (tr3; mean ~3.9Mb distant from nt). The manatee has presumptive functional *DRA* and *DRB* genes at the nt and tr2 region, respectively, this *DRB* gene has a 16 codon gap in exon 3 but was considered a functional gene since no stop codons were found. The *DR* subregion in manatee have several assembly gaps, which hinders a clearer definition of number of genes. Tr1 was only found in *Paenungulata* (i.e., *L. africana* and *T. manatus*). All afrotherians seem to have lost functional *DRB* from nt, whereas *E. edwardii* lack all *DR* loci in the region (Figure 2). Afrotherian species seem to maintain only one subregion with presumed functional *DRB* genes, either tr2 (Paenungulata, *O. afer*, and *Afrosoricida*) or tr3 (*E. edwardii*).

Pinnipeds possess *DRB* and *DRA* loci; walrus have an *in tandem* duplication of *DR* loci in the nt. The only *DRA* locus from Hawaiian monk seal has a 1-bp deletion in exon 2 leading to several stop codons, and therefore was annotated as a pseudogene. However, this species has a presumptive functional *DRB* gene. Pinnipeds (except the Hawaiian monk seal) also share with dog a *DRB* locus between *DQB* and *DOB* (termed “nt2” region) that seems to be functional in walrus and Weddell seal (Figure 3).

Cetaceans have one bona fide *DRA* gene and one to three *DRB* loci (Figure 4). The cetaceans and cattle share a one codon deletion on the first exon of *DRA* genes (Supplementary File S8). The cetacean *DRB* pseudogene in the class IIb region lies in a location similar to *DSB* in cattle (Figure 4). The position and direction of class IIb *DRB* pseudogenes are compatible to that of nt2 *DRB* in the non-inverted class II region of other mammals.

**Figure 5:**
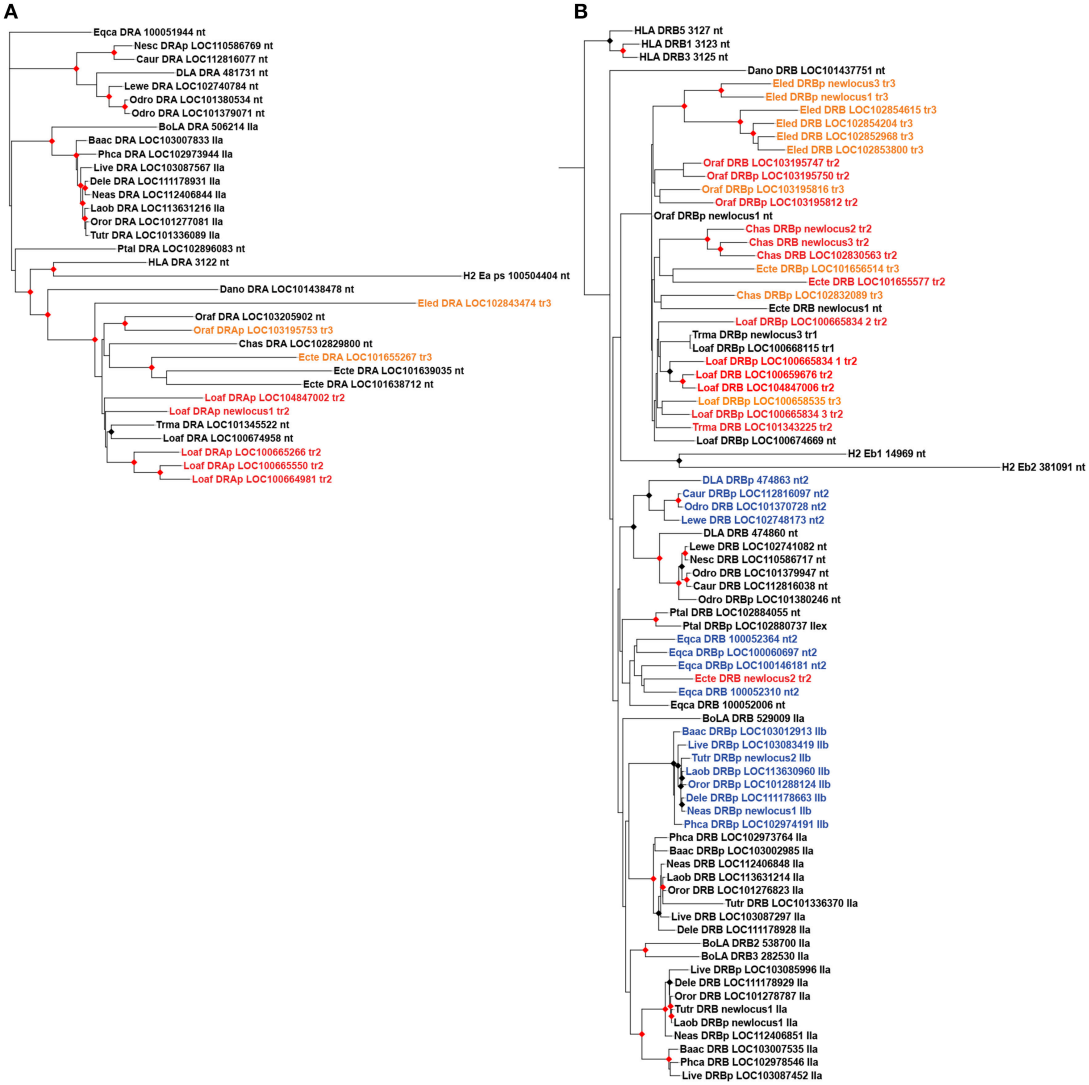
Maximum likelihood phylogenetic trees of *DR* MHC class II genes and pseudogenes. **(A)** *DRA* introns 1, 2, and 3 phylogeny; **(B)** *DRB* introns 1, 2, 3, 4, and 5 phylogeny. On the sequence positions: “nt” refers to not translocated; in blue, "nt2" refers to genes between *DQ* and *DO*, and IIb to genes located on class IIb region in Cetartiodactyla, in blue; “tr1” refers to translocated between C6orf106 and SNRPC; “tr2” between TULP1 and FKBP5, in red; “tr3” between CLPS and LHFPL5, in orange; “p” refers to pseudogenes. Black and red circles indicates >80 and >95% support values, respectively. *L. africana*, Loaf; *T. manatus*, Trma; *O. afer*, Oraf; *El edwardii*, Eled; *C. asiatica*, Chas; *E. telfairi*, Ecte; *D. novemcintus*, Dano; *B. acutorostrata*, Baac; *D. leucas*; Dele; *L. vexillifer*, Live; *N. asiaeorientalis*, Neas; *O. orca*, Oror; *L. obliquidens*, Laob; *T. truncates*, Tutr; *P. catodon*, Phca; *C. ursinus*, Caur; *N. schauinslandi*, Nesc; *L. weddelli*, Lewe; *O. rosmarus*, Odro; *P. alecto*, Ptal; *E. caballus*, Eqca; *B. taurus*, BoLA; *H. sapiens*, HLA; *M. musculus*, H2; *C. l. familiaris*, DLA.

*DRA* phylogenies formed well-supported clusters separating Carnivora, Cetartiodactyla and Afrotheria loci (Figure 5A). The afrotherian translocated loci from different locations did not form a well-supported cluster on the phylogenies; overall, *DR* loci grouped by species and not by genomic position on the phylogenetic trees. The only evidence of orthology from different afrotherian species occurred in the Paenungulata *DRA* nt genes (Figure 5). Carnivora formed 2 well-supported clusters in *DRB* phylogeny separating nt from nt2 sequences, although horse nt2 loci did not cluster with the Carnivora nt2 loci (Figure 5B). Similarly, cetacean IIb loci formed a well-supported cluster apart from nt2 loci from horse and carnivores (Figure 5B). Cetacean IIa loci formed two clusters separating most *DRB* pseudogenes from the genes, and therefore the ancestor of cetaceans probably had one *DRB* gene and one pseudogene.

#### *DQ* Loci

**Figure 6:**
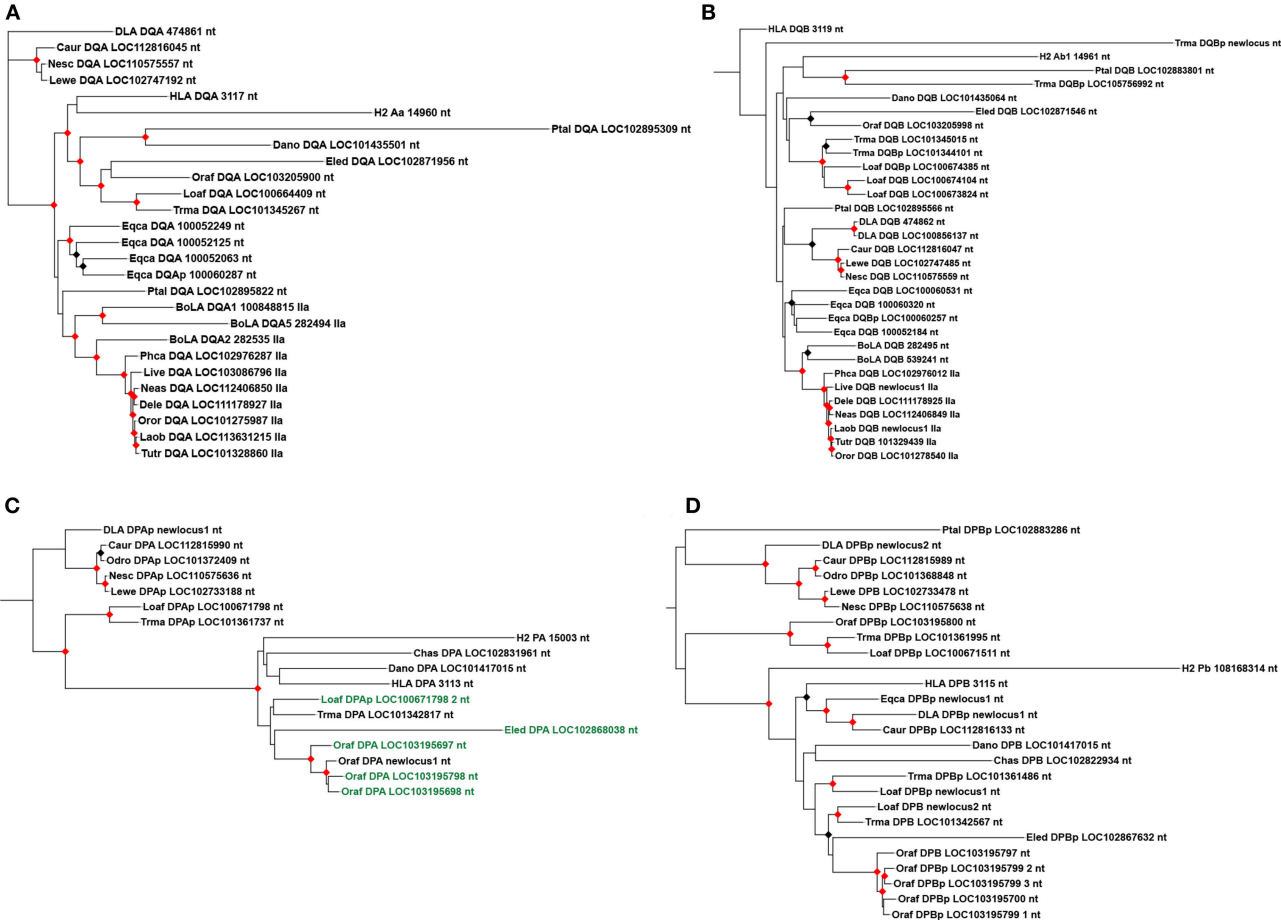
Maximum likelihood phylogenetic trees of *DQ* and *DP* MHC class II genes and pseudogenes. **(A)** *DQA* introns 1, 2, and 3 phylogeny; **(B)** *DQB* introns 1, 2, 3, 4, and 5 phylogeny; **(C)** *DPA* introns 1, 2, and 3 phylogeny; **(D)** *DPB* introns 1, 2, 3, and 4 phylogeny. In **(C)**, sequences with a 3-codon insertion on exon 3 are in green. “p” refers to pseudogenes. Black and red circles indicates >80 and >95% support values, respectively. *L. africana*, Loaf; *T. manatus*, Trma; *O. afer*, Oraf; *El edwardii*, Eled; *C. asiatica*, Chas; *E. telfairi*, Ecte; *D. novemcintus*, Dano; *B. acutorostrata*, Baac; *D. leucas*; Dele; *L. vexillifer*, Live; *N. asiaeorientalis*, Neas; *O. orca*, Oror; *L. obliquidens*, Laob; *T. truncates*, Tutr; *P. catodon*, Phca; *C. ursinus*, Caur; *N. schauinslandi*, Nesc; *L. weddelli*, Lewe; *O. rosmarus*, Odro; *P. alecto*, Ptal; *E. caballus*, Eqca; *B. taurus*, BoLA; *H. sapiens*, HLA; *M. musculus*, H2; *C. l. familiaris*, DLA.

The marine mammals have a similar *DQ* subregion, with at least a pair of *DQA* and *DQB* functional genes annotated in most species analyzed here. The manatee genome has one *DQA* gene and four *DQB* loci, although only one seems to be functional. Most afrotherians also have a single *DQA* gene, while the number of *DQB* loci varied across taxa. The only species analyzed with multiple presumptive functional *DQB* genes is *L. africana*. We could not find any *DQ* loci in the genome of *C. asiatica* and *E. telfairi*. Pinnipeds and cetaceans have a pair of *DQA* and *DQB* genes, however, no *DQ* loci was located in the minke whale genome, possibly due to the abundance of assembly gaps in the *DQ* subregion. *DQ* genes maintained overall conservation of exon length, with differences only in exon 1 of *DQB* (Supplementary Files S10, S11). Most manatee loci clustered with elephant sequences in the phylogenies (Figure 6), but one manatee *DQB* pseudogene clustered with *P. alecto* pseudogene, suggesting this pseudogene was present in the ancestor of eutherians (Figure 6B). Carnivora and Cetartiodactyla *DQA* and *DQB* genes clustered inside their groups; cetacean *DQA* is orthologous to BoLA-*DQA2* (Figure 6A).

#### *DP* Loci

We reannotated most *DP* loci, mainly due to difficulty in assigning exon 1 for *DPA* and exon 5 for *DPB*. Most *DPA* loci were annotated with a small exon 1, because the start codon seemed to have mutated (coding for a valine instead of methionine). Protein alignments are provided in Supplementary Files S12, S13. Among the marine mammals, the only species with a pair of presumptive functional *DPA* and *DPB* genes is the manatee (Supplementary File S3, Table 1); the manatee possesses three *DPA* and four *DPB* loci, but only two *DPA* and one *DPB* are presumptive genes. Inside Afrotheria, *O. afer* possesses four *in tandem* duplications of the *DP* loci, while *E. telfairi* lost all *DP* loci (Figure 2). Most pinniped's *DP* loci are pseudogenes, except one *DPB* gene in Weddell seal and one *DPA* in Northern fur seal (Figure 3); *Caur*-*DPA* lacks homology in the end of exon 4 and *Lewe*-*DPB* is a partial sequence including only exons three and four. Cetaceans lack *DP* loci altogether, with the exception of a remnant of a *DPB* pseudogene (homology only to the exon 3) found in minke whale (Figure 4).

Four *DPA* genes (three from *O. afer* and one from *E. edwardii*) and two pseudogenes (one from *O. afer* and one from *L. africana*) possess a distinctive three codon insertion in exon 3, which may be evidence of an ancestral form of *DPA* in the afrotherian lineage (Supplementary Files S3, S12). However, sequences with this insertion did not form a well-supported cluster in the phylogenies (Figure 6C). *DPA* showed signs of orthology in Paenungulata; *DPB* pseudogenes showed signs of orthology between Paenungulata and *O. afer* (Figure 6). The ancestor of eutherians seems to have had two *in tandem* duplications of DP loci, one with functional genes and the other with pseudogenes; the manatee has loci from both clusters (Figure 6). Most Carnivora loci grouped inside pseudogene clusters; the presence of *Caur*-*DPA* and *Lewe*-*DPB* inside this cluster of Carnivora pseudogenes suggests both may not be functional (Figure 6).

## Discussion

Here we report the organization of the marine mammal class II MHC and the first model for the evolution of this region in afrotherians, *sirenians* and pinnipeds, including species from the families Otaridae, Phocidae, and Odobenidae. We also expanded the number of class II MHC organization reports in cetaceans, including species from Mysticeti and Odontoceti lineages. We found that the manatee MHC includes the main classical mammalian class II genes while most *DR* loci were translocated—a feature manatee shares with the other afrotherians analyzed here. Both pinnipeds and cetaceans have presumptive functional *DQ* and *DR* genes, probably lost functional *DP* genes, and have one *DRB* locus lying next to *DOB*. These findings fill a gap in the study of marine mammal immunogenetics and eutherian MHC evolution, providing evidence of new chromosomal rearrangements events that led to changes in the organization of the mammalian MHC.

The afrotherian MHC is poorly studied as a whole—to our knowledge, the only reports on afrotherian MHC are two studies on the *DQA* polymorphisms in elephant and wooly mammoth (42, 43). Based on genomic resources analyzed here, the manatees share with other afrotherians a unique *DR* translocation separating it from the core class II region. Despite manatee genes being distributed over four main scaffolds, all class II MHC sequences, including translocated *DR* loci, presumably lie on the same chromosome, based on other afrotherian class II regions and data from chromosome painting in manatee (44). The similarity between the manatee and elephant class II organization suggests that elephant may serve as a model for understanding the manatee MHC function. Antigen presentation in manatee presumably uses *DR, DQ*, and *DP*, with evidence of *DQ* duplications. Future research addressing the expression and polymorphism of class II genes in both species is needed. Afrotherians also have other unique features: a three-codon insertion on exon 3 in some of the *DPA* loci and deletion of *DQ* and *DP* loci during Afrosoricida (tenrec and golden mole) evolution. It is important to notice that tenrec (*E. telfairi)* seem to have one of the simplest mammalian MHC class II regions. We found three *DRA* genes and only one *DRB* gene on the tenrec assembly (Figure 2; Supplementary File S3), which may represent a mammalian “minimal essential” MHC class II, like in chickens—with only two classical class II genes, coding the alpha and beta peptides (45).

Despite several reports on class II gene polymorphisms in pinnipeds, to our knowledge this is the first analysis of MHC structure in this clade. The class II MHC of pinnipeds is similar to the DLA organization (46). The lack of a pair of presumed functional *DPA* and *DPB* genes in pinnipeds suggests their MHC may function similarly to cetaceans—using primarily *DR* and *DQ* molecules—and may provide opportunities to investigate convergence in class II evolution in both clades. Most pinnipeds also have an inverted *DRB* locus between *DQB* and *DOB*, which is not only present in carnivores such as dogs (46) and cats (47) but also in horses (48). This inversion event is thought to have occurred in the ancestor of Laurasitheria (48) and this *DRB* may be functional in walrus and Weddell seal.

Despite several studies focused on class II gene polymorphism in cetaceans, their MHC structure was only recently characterized (26). Most cetaceans analyzed here and the previously reported Yangtze finless porpoise MHC (26) have one *DRA* and two *DRB* loci in class IIa, a *DRB* pseudogene in class IIb, a single *DQ* pair, and lack *DP* and *DY* genes. On the other hand, cattle have *DYA, DYB, DSB*, and duplicated *DQ* genes; therefore using cattle as a model for cetacean immunogenetics should be cautionary until further characterization of expression and MHC haplotype variation in cetaceans. Notably, the only *Mysticeti* species analyzed here lacks *DQ* loci in its assembly, probably due to the large assembly gaps in this region, since there are reports of *DQ* polymorphisms in baleen whales (16, 19).

The difficulties of assembling the MHC region is widely known, due to extensive variation in gene sequence and haplotype composition of multigene families. However, due to increasing availability of good non-model species genomes, researchers have started using publicly available genomes to analyze the MHC region (49–56). Despite the challenges, to our knowledge, there are no reports that such difficulties resulted in artifactual chromosomal rearrangements of MHC loci, such as the translocations seen in Afrotheria. This mutation event is supported by the fact that two different sequencing technologies (long-reads from Sanger and short-reads from Illumina) and two different *de novo* assembly algorithms [ARACHNE and ALLSPATH (57, 58)] resulted in the same translocated subregions across the afrotherian genomes. Thus, if the translocation was an assembly artifact, the same misassembly would have to be repeated six times independently with datasets from different species, generated with different sequencing methodologies and different assembly algorithms. It is important to note that no other mammals analyzed here or elsewhere had similar events. The MHC organization of the other analyzed marine mammals were highly consistent with the organization of their eutherian lineages. Similarly, the deletion of *DQ* in Afrosoricida is supported by the evolutionary relationship of *C. asiatica* and *E. telfairi*—a deletion shared by two independent assemblies of animals from the same lineage and by the overall reduction in MHC size in the species scaffolds especially in the *DQ/DP* subregion. Another way to provide physical evidence for the translocation or deletion would be to use fluorescent *in situ* hybridization or sequence BAC libraries containing MHC genes, which was beyond the scope of the present study.

The *DR* phylogenies clustered sequences by species, although we expected that all translocated loci across afrotherians would form a well-supported cluster in the phylogenies, separated from non-translocated sequences. *DRB* loci in the nt2-IIb region and *DPA* with a three-codon insertion also did not cluster in the phylogenies. Previous research, using class II MHC genes from laurasitherians, also had similar results (48). Orthologous relationships were particularly observed inside Cetacea and Carnivora in which species diverged < 60 Ma (40, 41). A better resolution of orthology among translocated loci would probably be achieved using less divergent taxa, since the clades analyzed here diverged early in afrotherian evolution. For instance, one of the closest pair of species studied here is the African elephant and the Florida manatee, with estimates of 70~65 million years of divergence (39, 59). The non-translocated *DRA* from manatee indeed clustered with the non-translocated elephant homolog, but the lack of other sequences (i.e., translocated *Trma*-*DRA* and non-translocated *Trma*-*DRB*) presently hinders a more comprehensive analysis.

**Figure 7:**
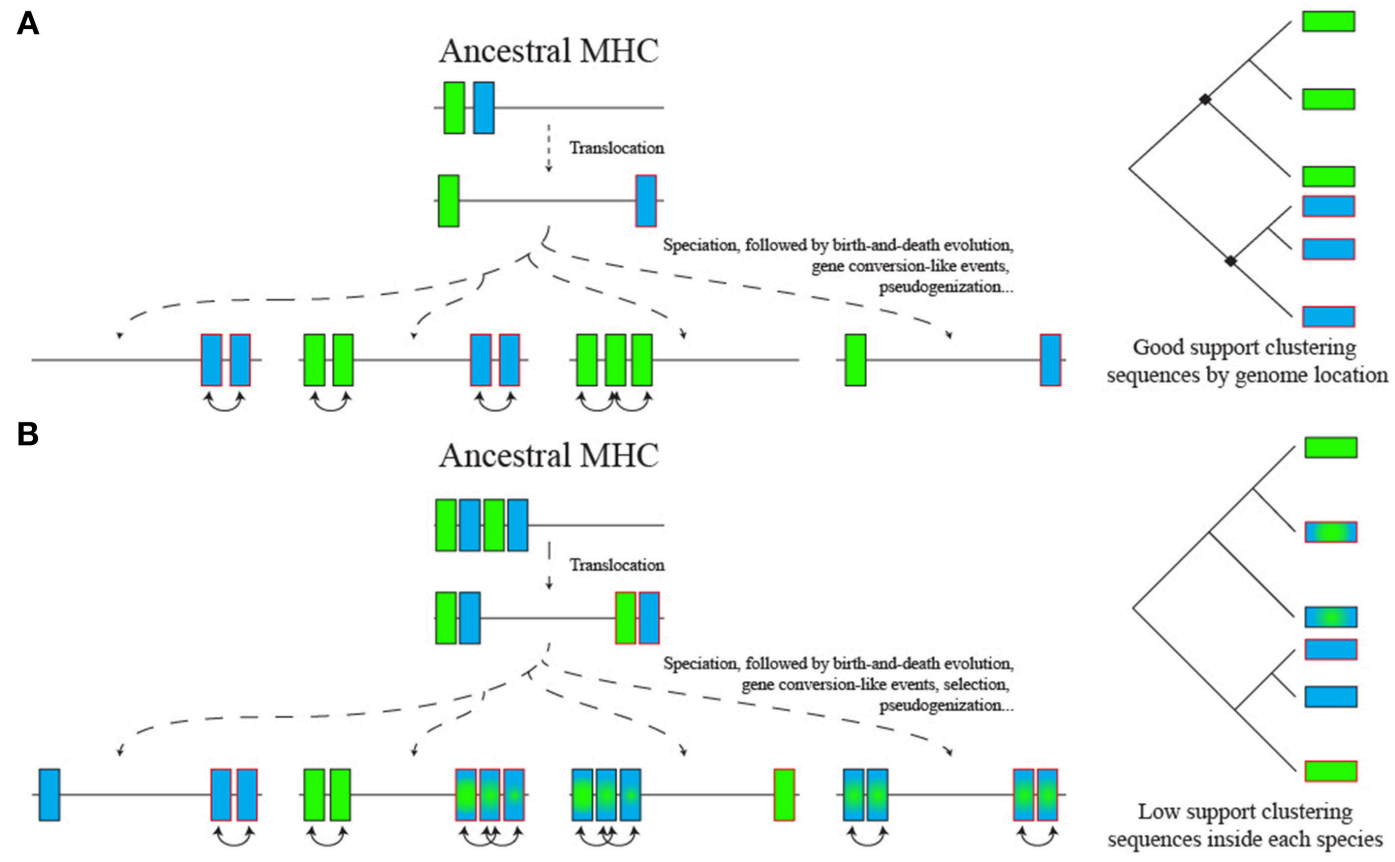
Two scenarios for ancestral MHC class II region evolution and impact on phylogenetic analysis. **(A)** A simple model with only two paralogs separated by a translocation event. **(B)** A complex model with *in tandem* duplication of two loci separated by a translocation event. In the first scenario, translocated genes from current species would cluster together in the phylogeny, while in the second scenario a birth-and-death model of evolution, including gene conversion-like events and differential loss of tandemly duplicated loci, results in no true orthologs across distantly related taxa. Selection acting on both translocated and non-translocated regions can also blur the phylogenetic signal in both scenarios both for exonic and intronic sequences.

In a simplistic scenario of the ancestral Afrotheria MHC, the translocated loci would diverge, and a phylogenetic analysis would separate loci from both regions (Figure 7A). However, in a realistic scenario, birth-and-death evolution (see below), natural selection, occasional gene conversion-like events and recombination may blur the evolutionary relationship among loci (Figure 7B). Those evolutionary processes may result in no true orthologs for MHC genes between distantly related taxa (60, 61). Similar processes may also explain why nt2 *DRB* loci and *DPA* loci with a three-codon insertion did not form a well-supported group in the phylogeny (since it is unlikely that both are cases of homoplasy).

The birth-and-death model of evolution for gene families—in which duplication, deletion and pseudogenization of genes lead to expansion and contraction of gene families (61)—affects both MHC class I and II genes but is more pronounced in the former, which usually results in lack of orthology when comparing animals from different families/orders (60). It has been proposed that the class I region evolves faster in eutherians due to its separation from the antigen processing genes (62); in addition, teleost classical class II genes are separated from the rest of the MHC and evolve similarly to eutherian class I genes (62). The variation in the number of *DR* loci in the afrotherian translocated regions suggests that the separation of *DR* loci from the class II region may have allowed genes to evolve faster, which could account for the loss of orthology seen in the phylogenies. Even though the translocation separated two DR subregions, the coded proteins still must form a functional heterodimeric class II protein that can interact with the TCR/CD4 complex of T lymphocytes. Thus, alpha and beta *DR* genes may coevolve and converge irrespective of their position in the genome, which again may impact phylogenies.

The clustering of genes related to antigen processing and presentation in the MHC and their conserved organization in eutherians is thought to be of functional importance (63). In mammals, early evidence of disruption in this organization was found in ruminants, in which an inversion split their class II region into two subregions (64, 65), an event now known to have taken place in the Cetartiodactyla ancestor (26). Since then, other studies revealed additional events disrupting this seemingly conserved organization: an inversion on distal class I region and loss of functional *DQ* and *DP* in felines (66); loss of *DR* in mole rats (67); disruption of the MHC organization and pseudoautosomal localization in monotremes (68); and several rearrangements in class I and II regions in wallaby (69). Those reports, including ours, provide opportunities to investigate how the MHC function evolves in different genomic landscapes and may challenge the functional importance of conserving the MHC organization (70). We also note that no afrotherian or xenarthran mammals had their entire MHC organization characterized, therefore our results show that the separation of class I and class II genes took place in the ancestor of all living eutherians after the split with marsupials [~170 million years ago (59, 71)], as suggested by Belov et al. (72).

The comparative study of the MHC in marine mammals may address how each lineage dealt with unique pathogen pressures in marine environments with the proposed distinct MHC class II genomic organization: the sirenians, with three classical gene families and the presumptive afrotherian translocated *DR* loci; the pinnipeds, with two classical gene families and inverted *DRB* loci; and the cetaceans, with two classical gene families and the cetartiodactyl inversion separating class IIa and IIb. Due to the lack of gene expression studies, the presumed annotation of genes and pseudogenes presented here should be interpreted with caution. Therefore, our results mandate future studies focusing upon in depth characterization of the structure, function and expression of the MHC as well as other important immunogenetic systems—such as TLR, Ig, and TCR, already in progress for some lineages and genes (73–78)—in the three marine mammal lineages. Direct sequencing and transcriptomic data will help clarify which sequences are functional, the degree of polymorphisms and any functional specialization of duplicated or translocated loci. Future research may use data provided here to carefully design amplification schemes that target canonical, translocated, inverted or IIb *DRB* loci.

Taken together, our results indicate a unique class II MHC architecture in each major marine mammal lineage. The evidence presented here also shows a sequential loss of two classical class II genes during Afrosoricida evolution, which may have resulted in the simplification of the class II region in *E. telfairi*, with only one classical class II protein encoded. Those results point to the separation of MHC class I and II regions in the ancestor of all living eutherians and reiterates the challenges to uncovering evolutionary relationships between MHC genes in distantly-related taxa. The occurrence of rearrangements in the mammalian MHC suggests this highly conserved system may be more malleable than once thought.

## Author Contributions

AS, LS, MC, and MS designed the study. AS performed all analysis and prepared figures. AS, BB, and TD reannotated the class II genes and performed dot plot analysis. AS and TB performed the phylogenetic analysis. LS, MC, and MS supervised all analysis. AS, BB, LS, and MC prepared the manuscript. All authors reviewed and approved the manuscript.

### Conflict of Interest Statement

The authors declare that the research was conducted in the absence of any commercial or financial relationships that could be construed as a potential conflict of interest.
